# Psychiatric consequences and issues of long COVID on patients with prior psychiatric comorbidities: a scoping review

**DOI:** 10.3389/fpsyt.2023.1181767

**Published:** 2023-06-07

**Authors:** Francesca Hovagemyan, Adeline Dugerdil, Axelle Braggion, Luc Mallet, Antoine Flahault

**Affiliations:** ^1^Institute of Global Health, Faculty of Medicine, University of Geneva, Geneva, Switzerland; ^2^Department of Mental Health and Psychiatry, University of Geneva, Geneva, Switzerland; ^3^Univ Paris-Est Créteil, DMU IMPACT, Département Médical-Universitaire de Psychiatrie et d’Addictologie, Hôpitaux Universitaires Henri Mondor—Albert Chenevier, Assistance Publique-Hôpitaux de Paris, Créteil, France

**Keywords:** long COVID, SARS-CoV-2, mental health, psychiatric symptoms, psychiatric history

## Abstract

SARS-CoV-2 is a growing field of research and mental health in long COVID is one of its interesting domains. This scoping review aims at studying the outcomes of mental health in patients already known for psychiatric illness. This was done by researching the literature in two databases (Embase and PubMed) for articles studying mental health consequences of long COVID in patients already known for psychiatric history. Eleven studies were included. 6/11 studies found an effect of long COVID, with varying severity of outcomes studied, with either a worsening in length or severity. 4/11 did not find any correlation between worsening symptoms and psychiatric history. The methods for assessing which psychiatric symptoms to include and how to determine prior history were heterogeneous, making direct comparison sometimes difficult. The data seem to show worse effects of long COVID on mental health of patients with prior mental illness, with limitations regarding the heterogeneity of the studies’ designs and focuses. It also highlights how neglected this population of patients is in the current state of research.

## 1. Introduction

We have yet to fully grasp the impact of the COVID pandemic on society and medicine, and the more we try to understand the consequences it may have had on the general population, the more we are aware of the enormous changes it has caused. The societal impact of COVID on the mental health of populations is getting an increasing amount of attention, showing that the general population is affected, but also the population already burdened by mental health problems (1, 2). Regarding the physical infection itself, an increasing number of published articles show that COVID-19 infection triggers cytokine release in the brain, causing neuro-inflammation (3). This is the presumed cause of some neuropsychiatric occurrences that were described in scientific literature, including catatonia (4), episodes of mania (5), psychosis (6), or even Cotard syndrome (7) in individuals who were not known for any kind of prior psychiatric history (8, 9). We know that long COVID can also burden with psychiatric diseases patients that were previously healthy, with the emergence, for example, of fatigue, sleep disorders, anxiety, and depression (10), with around a fifth of patients concerned by depression and almost a quarter by anxiety (11). The severity of the mental health problems seems also to be linked to the gravity of the infection (12). The World Health Organization defines long COVID as “the continuation or development of new symptoms 3 months after the initial SARS-CoV-2 infection, with these symptoms lasting for at least 2 months with no other explanation” (13). Nevertheless, in this review, we will use the expression “long COVID” referring to “signs and symptoms that continue for more than 4 weeks and can be attributed to COVID-19 infection” (14). We will also use “post-acute COVID syndrome,” “post-acute sequelae of COVID” (PASC) or “post-COVID” as interchangeable expressions, as differences exist between the terms, but are not relevant for this review. For example, studies have reported that SARS-CoV-2 infection could lead to modified pharmacodynamics and therefore drug toxicity in the treatment of bipolar disorder (15). The treatment of severe COVID-19 in itself can also be composed to some extent of steroid administration, which could rarely cause acute psychiatric effects when used in this indication (16). Patients with somatic chronic diseases also seem to experience mental illness more frequently, especially those who feel they have less symptom control and an impaired quality of life (17). Dantzer et al. (18) further argue that in other chronic diseases known to cause systemic inflammation (systemic infections, autoimmune diseases, and cancers) the immune response in the brain can lead to depressive states. But what about patients with a history of psychiatric disease or comorbidities predating the SARS-CoV-2 pandemic? What mental health issues does long COVID bring up in patients with pre-existing psychiatric comorbidities? In this scoping review, we aim to evaluate the present state of research of the possible effect of long COVID on the mental health of patients with prior psychiatric comorbidities or a previous history of psychiatric illness. This scoping review aims at focusing on the psychiatric effects of the long haul infection itself, and will not look into the impact of the societal aspects of COVID-19, or COVID-19 policies on the general population’s or psychiatric patients’ mental health.

## 2. Materials and methods

### 2.1. Search strategy

**Figure 1:**
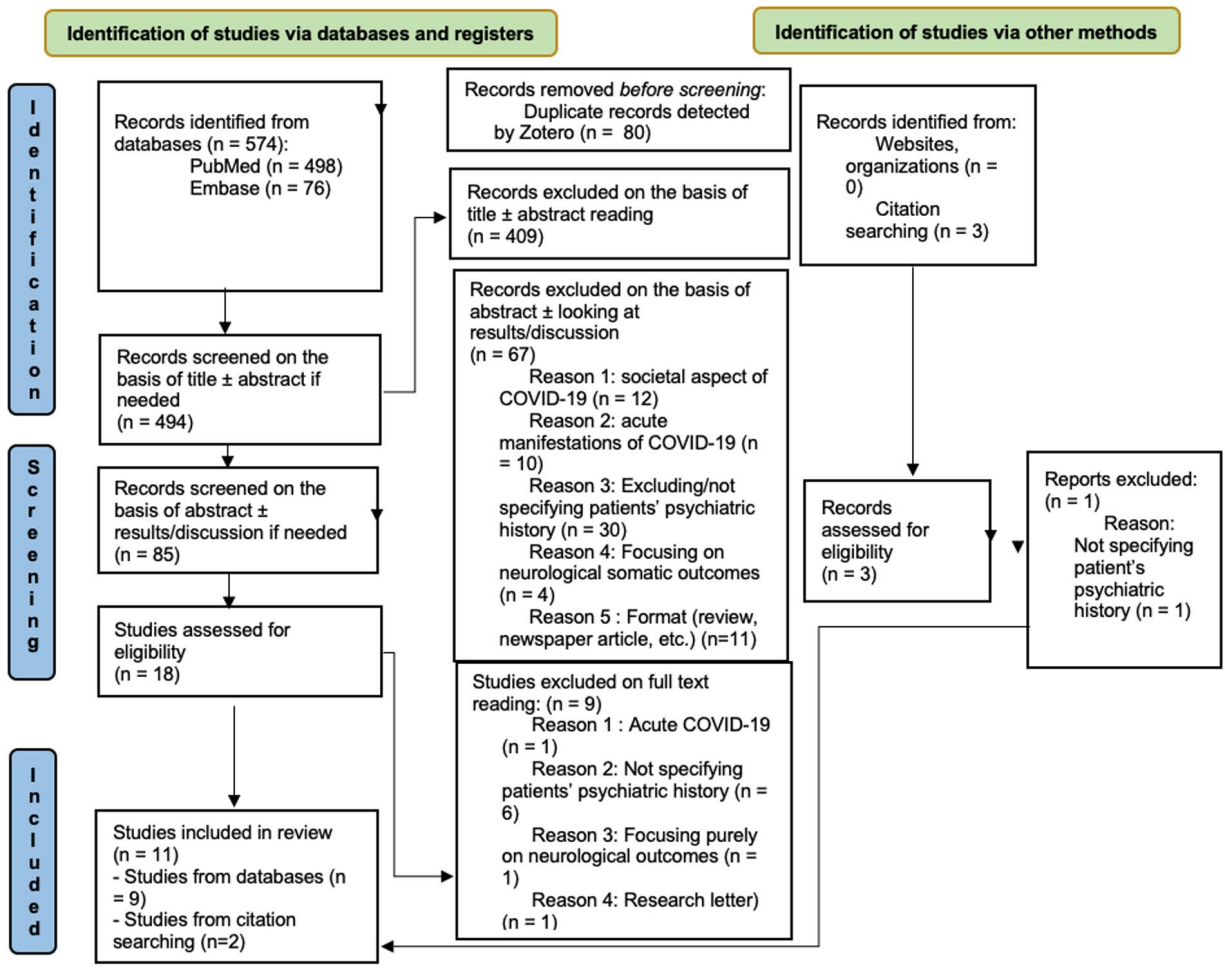
PRISMA-chart.

A preliminary search of the Prospero database, as well as Google Scholar, PubMed, and Embase was previously performed and no previous scoping reviews or meta-analysis were found on the subject. The process of selecting the included articles will be summarized in the PRISMA chart (Figure 1). We performed a scoping review using the PRISMA-ScR guidelines (19). The search databases Embase and PubMed were browsed between the 16th and the 18th of November 2022. We included original research articles, both prospective and retrospective observational studies, case series, and case reports. Meta-analyses were also considered but none was found. We did not include reviews in our research as we wished to focus on original research articles. We performed one round of research in the two databases, with two research queries, essentially containing the same words but adapted to the specificities of each database. The research queries are enlisted in Appendix A.

### 2.2. Inclusion and exclusion criteria

For a better quality of review, we restricted our research to articles published between January 1, 2022 and November 18, 2022 (date of final database search), written in English and focusing or having collected substantial data on patients having prior psychiatric history with a recent COVID-19 infection. The minimum timeframe between infection and study, or study follow-up, was 4 weeks post-infection. We focused on records concerned with psychiatric illnesses as broadly as possible, including: depression, anxiety, obsessive–compulsive disorders (OCD), post-traumatic stress disorder (PTSD), schizophrenic and psychotic syndromes, bipolar disorder, eating disorders, and cognitive impairment. We included articles discussing both hospitalized patients and outpatients. We excluded, from our first round of research, articles written outside of the timeframe defined above, written in any language other than English, as well as articles on a more sociological or political aspect of the SARS-CoV-2 pandemic and its effect on mental health: for instance, the impact of lockdown policies on mental health or lessened well-being spawned by the changes in society during COVID-19 times.

Studies only based on interventional aspects were excluded, as they focused on the outcomes of the intervention and did not describe the context. We also excluded papers focusing on the somatic and neurological outcomes of long COVID or on sudden psychiatric manifestations (catatonia, Cotard syndrome, and mania) during acute infection in previously healthy patients. We also chose to disregard papers focusing exclusively on fatigue, “brain fog” and sleep disturbance outcomes. Firstly, those aspects are not specific to psychiatry and secondly, we consider this aspect to be such a relevant and voluminous subject that it needs to be treated separately so as not to cancel other, smaller and less frequent manifestations of (neuro-)psychiatric long COVID. Nevertheless, if the subject of fatigue was treated in combination with other aspects that intersected our research area, we did not exclude the article. Lastly, we excluded articles regarding patients deceased from acute SARS-CoV-2 infection, as we wanted to study the consequences in the middle-to-long-term of the post-acute syndrome.

During the screening process, we read articles that were not ultimately selected but that cited other noteworthy articles. As we read these articles, two of them (*n*° 10 and 11) seemed to fit the subject of the present research and were widely cited in papers on mental health and long COVID. We therefore decided to include the two articles despite their publication outside the chosen timeframe.

### 2.3. Data extraction

The articles found with the research strategy described above were uploaded on Zotero 6.0.16. Duplicates were automatically detected and manually deleted upon verification. The articles were then screened on the basis of title relevance to the subject. In cases of ambiguity, the abstract was read, reducing the number of articles selected for a full text reading. These articles were recorded in an Excel program (version 16.16.5) and their abstracts were read for further selection, with examinations of results or discussion sections when in doubt. The selected articles were read in full text and the data extracted. They were then reviewed by a second reader, who also reviewed the categorization table. Any disagreement was discussed and referred to a third reader if necessary.

## 3. Results

As can be seen on the PRISMA chart (Figure 1), the first search of the databases returned 574 results (498 in PubMed and 76 in Embase). On the basis of titles, we removed 489 articles. After reading the abstracts, 18 articles were selected, and nine more were excluded on full text reading, leaving nine studies for data extraction. Adding the two studies found by other means, 11 studies were finally included at the end of the screening process.

The data extracted from the selected articles is regrouped in Tables 1, 2. For legibility purposes, the articles are referenced in all tables by number. A separate bibliography can be found for the articles used for the scoping review, with full references (Appendix B). Among the 11 studies included, 3 (27.3%) were retrospective studies, 3 (27.3%) were cross-sectional studies, 2 (18.2%) were case reports, one was a descriptive study (9.1%), and 2 (18.2%) were cohort studies. The total number of participants (all studies combined) was 9,137, of which 1,502 (16.4%) were patients with a psychiatric history (PWPH). It should be noted that one study (*n*°2) did not specify how many PWPH were among the participants. If including case reports—where the rate of PWPH was 100%—the mean rate of PWPH in each study was 37.8%. Since we think the inclusion of the case reports introduces a bias in our data, we excluded them from the numbers in Table 3: this makes a rate of 24% of PWPH included in the studies of this review.

**Table 1:** Pertinent data extraction of articles reviewed.

#	Type of document	Location of study	Psychiatric premorbid history	Aim of study	Population	Sample size	Time between infection and study	Pertinent results
1	Retrospec-tive study	IRCCS, Milan, Italy	PDWS “RCD+” (specified)	To describe the evolution in cognitive fields in patients RCD+ vs. RCD-	COVID-post-infectious patients	54 patients, 37 RCD+ of which 5 (9.26%) with psychiatric history	127.65 days ± 114.11 (21–422) in RCD+ and 77.59 ± 33.84 (22–154) (~4 months)	Worse cognitive decline in patients RCD+ not found when looking at adjusted value.
2	Retrospec-tive study	IRCCS, Milan, Italy	PDWS *or neurological disease* “Neuro+” (unspecified)	Investigation of premorbid state or background and its eventual correlation with worse psychiatric sequelae.	COVID-19 recovered inpatients	152 patients, no specific data on PWPH	88.31 days ± 76.11 (7–422) for Neuro+, 74.75 ± 32.44 (26–186) for Neuro- (~3 months)	Higher pathological score in psychometric scales for Neuro+ patients but too many confounding factors do not allow to draw definitive conclusions
3	Web-based cross sectional survey	Japan	DD, BP, AD, AA, and others	Impact of psychiatric history prior to infection on the severity of psychiatric symptoms after contracting the COVID-19	Survey panel of a major national survey agency	6,016 participants, of which 1,067 (17.7%) with psychiatric history	1, 3, and 6 month after time of infection	PWPH have a less favorable evolution over time of anxiety and depression than patients without a psychiatric history, likely because of a higher basal neurological inflammation and lessened resilience capacity
4	Cohort study	Austria and Italy	DD, AD, and SD	Exploring COVID-19 disease course as well as physical and mental recovery, taking into account demographics, psychosocial stress, socioeconomics, and premorbid history.	Adult non-hospitalized COVID-19 convalescents	2,053 participants, of which 199 (9.69%) with psychiatric history	At least 28 days after diagnosis, mean in Austria: 79 days (40–180), in Italy: 96 days (60–140) (1–3 months, μ = 2)	No effect of prior mental illness on post-infectious first onset anxiety and depression when compared with the group without psychiatric history
5	Case report	Leuwen, Belgium	ED (Bulimia nervosa)	Impact of smell and taste disorder on bulimia nervosa	Female of 20 years burdened with Bulimia nervosa	One patient (100% with a psychiatric history)	6 months	Change in trigger for BE episode, prior to infection = stress, post-COVID = craving instead
6	Not specified, cohort study according to methodo-logy	Columbia CUIMC, United States	DD, AD, and PTSD (processed together)	Assessment of cognitive symptoms 3 months after COVID-19 infection, taking into account cofactors, as current depression, prior mental illness, etc.	Patients treated for COVID-19 at CUIMC ED or IW, out 26.03–27.05.2020	153 participants, of which 23 (15%) with psychiatric history	3.68 (median) months post-COVID discharge	Psychiatric history is a risk factor for cognitive decline 3 months after COVID-19 infection (aOR 4.9)
7	6-year longitudi-nal case report	Canada	BD	Exploring the evolution of cognitive functioning after COVID-19 infection over three months in a patient known for bipolar disorder	78-y.o. F with a long history of BD	One patient (100% with a psychiatric history)	3 months	Significative worsening of cognitive state after COVID-19 infection in every field-tested except verbal intelligence, short-term auditory attention span, confrontation naming, expressive vocabulary, single word reading, and object perception
8	Descripti-ve study	USA (not specified, deduced from article reading and location of authors)	AD, DD, PTSD, BD, and AA	(1) Description of cognitive functions in PASC; (2) Evaluate the relationship between PASC and demographics, disease severity, premorbid diagnoses, and current emotional distress on cognitive functioning	Post-COVID patients referred for neuropsy-chological evaluation	49 participants, of which 28 (57.1%) with psychiatric history	6 months	No impact revealed on cognitive measures of premorbid psychiatric history
9	Retrospec-tive study	NYMC, New York, United States	AD, DD, and adjustment disorder	Evaluation of PASC on a neuropsychiatric point of view, including role of past psychiatric history	Patients who had COVID-19 illness and received a psychiatric consultation in the outpatient setting	30 patients, of which 17 (56.7%) with a psychiatric history	6 months average	No effect detected of psychiatric history for new symptoms of AD, DD, SD following COVID-19 illness. Interpretation of symptoms as pure complications of infectious disease, without regard to past psychiatric history.
10	Cross-sectional study	IRCCS, Milan, Italy	PDWS	Psychopathological impact of COVID-19 after one month, taking into account risk factors	Surviving COVID-19 patients with an ED admission (± hospitalization)	402 patients, of which 106 (26.4%) with a psychiatric history	1 month	PWPH have a worse outcome in all current psychopathology measures than patients without a history
11	Cross-sectional study	IRCCS, Milan, Italy	PDWS	Psychopathological impact of COVID-19 after three months, taking into account risk factors	Surviving COVID-19 patients with an ED admission (± hospitalization)	226 patients, of which 55 (24.3%) presenting criteria for a major psychiatric disorder	3 months	Worse persistence of depressive symptomatology in PWPH and higher level of symptoms

AA, alcohol abuse; AD, anxiety disorder; BP, bipolar disorder; CUIMC, columbia irving medical center; DD, depressive disorder; ED, eating disorder; ED; emergency department; IRCCS, Istituti Clinici Scientifici Maugeri of Milan; IW, inpatient wards; NYMC, New York Medical College; OCD, obsessive–compulsive disorder; PASC, post-acute sequelae of COVID-19; PD, personality disorder; PDWS, psychiatric disease without specification; PTSD, post-traumatic stress disorder; PWPH, patients with psychiatric history; RCD+ (Aiello et al.): patients at risk for cognitive deficits; SD, sleep disorder; SUD, substance use disorder; *t*, threshold.

**Table 2:** Studies included, sorted by neuropsychiatric outcome.

#	Prior psychiatric history	Way of finding information	Neuropsychiatric outcome studied	Psychometric scale used for psychiatric evaluation	Results
1	PDWS (at least one neurological/psychiatric condition)	Retrieval from medical records	Cognitive functions	MMSE, ACE-R (y c. ACE-R-OA, ACE-R-M, ACE-R-F, and ACE-R-*VS*), FAB, and AM	No differences RCD+/− if adjusted values
6	Depression, anxiety, and PTSD	Self-report	Cognitive functions	Cognitive Change Index (3 questions ≠ formal), PCL-5 cued to COVID-19 illness, PHQ-8	No conclusions drawn
7	Bipolar disorder	Long known patient (psychiatric follow-up 6 years prior to infection)	Cognitive functions	Standardized scores in the memory domain and standardized scores in non-memory domains (see under)	Significative, durable worsening cognitive functioning
8	Depression, anxiety, PTSD, bipolar disorder, and alcohol abuse	Retrieval from medical records	Cognitive functions	Clinical interview + standardized scores for cognitive assessment (see under)	No impact of premorbid psy. history
3	Depression, anxiety, bipolar disorder, and alcohol abuse	Self-report (questionnaire)	Depression and anxiety	PHQ-9, GAD7	Less favorable evolution for PWPH
4	Depression, anxiety, and sleep disorder	Self-report (questionnaire)	Depression	PHQ-4	No effect of psychiatric history on depression outcome
9	Depression, anxiety, and adjustment disorder	Self-report	Depression, anxiety, (fatigue and memory: not presented in results)	PHQ-9, GAD-7, C-SSRS, FSS, MOCA, and psychiatric clinical interview	No effect of psychiatric history on depression and anxiety outcomes
2	Previous neurological or psychiatric diagnosis (anxiety, mood or psychotic disorder)	Retrieval from medical records	Depression, anxiety, fatigue, PTSD, and cognitive functions	PHQ-9, GAD-7, IES-R, MMSE, and MoCA	Higher pathological results but not interpretable
10	PDWS	Self-report (questionnaire)	Depression, anxiety, PTSD, sleep, and OCD	Psychiatric clinical interview + IES-R, PCL-5, ZSDS, BDI-13, STAI-Y, MOS-SS, WHIIRS, and OCI	Worse outcomes on all psychopathology measures for PWPH
11	PDWS	not specified but same design as #10: probably self-report	Depression, anxiety, PTSD, sleep, OCD, and cognitive functions	Psychiatric clinical interview + IES-R, PCL-5, ZSDS, BDI-13, STAI-Y, WHIIRS, and OCI ± BACS	Worse persistence of depression in PWPH
5	Eating disorder (bulimia nervosa)	Long known patient + self-report of symptoms	Eating disorder	EDE-Q score, DASS-42	Change in bulimia triggers

ACE-R, Addenbrooke’s cognitive examination—Revised (-OA, orientation and attention; -M, memory; -F, Fluency; and -*VS*, visuo-spatial abilities); AM, attentional matrices; BACS, brief assessment of cognition in schizophrenia (cognitive assessment); BDI-13, 13-item beck’s depression inventory (assessment of depression); C-SSRS, columbia suicide severity rating scale; DASS-42, depression anxiety stress scale; DASS-42, depression anxiety stress scale; EDE-Q score, eating disorder examination questionnaire; FAB, frontal assessment battery; FSS, fatigue severity scale; GAD-7, general anxiety disorder-7 (assessment of anxiety); HVRTL, hopkins verbal learning test revised; IES-R, impact of event scale—Revised (assessment of PTSD); MMSE, mini mental state; MoCA, montreal cognitive assessment; MOS-SS, medical outcomes study sleep scales (assessment of sleep disorder); NAB, neuropsychological assessment battery; OCI, obsessive–compulsive inventory (assessment of OCD); PCL-5, PTSD checklist (DSM-V); PHQ-n, patient’s health questionnaire (assessment of depression); RCFT, rey complex figure test; STAI-Y, state–trait anxiety inventory form Y (assessment of anxiety); WAIS-IV, Wechsler adult intelligence scale-4 (cognitive assessment); WCST, Wisconsin card sorting test (cognitive assessment); WHIIRS, women’s health initiative insomnia rating scale (assessment of sleep disorder); WMS-IV, wechsler memory scale-4 (cognitive assessment); WRAT-4, wide range achievement test-4 (cognitive assessment); and ZSDS, Zung self-rating depression scale (assessment of depression). Detail item 7: psychiatric evolution evaluation: Standardized scores in the memory domain. KBNA, Kaplan Baycrest neurocognitive assessment; WMS-IV LM, Wechsler memory scale, Fourth Edition, Logical Memory. Standardized scores in non-memory domains: FSIQ, full scale intelligence quotient; KBNA, Kaplan Baycrest neurocognitive assessment; TOPF, test of premorbid functioning; VOSP, visual object and space perception battery; WAIS-IV, Wechsler adult intelligence scale, Fourth Edition; WASI-II, Wechsler abbreviated test of intelligence, Second Edition. Detail item 8: psychiatric evolution evaluation: Cognitive assessment: WRAT-4, wide range achievement test-4; WAIS-IV, Wechsler adult intelligence scale-4; WCST, Wisconsin card sorting test; WMS-IV, Wechsler memory scale-4; HVLTR, Hopkins verbal learning test revised; RCFT, Rey complex figure test; NAB, neuropsychological assessment battery; verbal fluency and animal fluency; stroop word and color; Grooved; BDI-II, beck depression inventory; and BAI, beck anxiety inventory.

**Table 3:** Mean rates of patients with a psychiatric history (PWPH) in studies included.

Mean percentage of PWPH	7.8%
Percentage of PWPH in total	16.4%
Mean percentage of PWPH *without including case reports*	24%

Three studies (3/11, 27.27%, studies *n*°3, 5, 7) had the effects on mental health of PWPH in long COVID (see Table 1 under “Aim of study”) as their main subject.

The duration of follow-up or window of time between infection and study was of a minimum of 1 month as defined above. The mean duration was of 3.97 months, with a maximum of 6 months follow-up.

Six studies (6/11,54.55%) found at least some effect of prior psychiatric history on neuropsychiatric recovery from COVID-19 at the time of follow-up, with mental health issues acting as a predictor for either worse adverse outcomes in mental health (*n*°3, 6, 7, 10, and 11), longer recovery (*n*°3), or a modification of the trigger of symptoms of the illness, without it being a better or worse outcome (*n*°5). One study (*n*°2) stated that its design did not permit the authors to draw precise conclusions on the matter.

Four studies (4/11, 36.36%, *n*°1, 4, 8, and 9) did not find any effect of psychiatric history on the mental health outcomes they looked at, with one study (*n*°9) going further and stating that interpretation of psychiatric symptoms following a COVID-19 infection should be interpreted as a complication of the infectious disease, without regard to prior mental health of the patient.

All studies we examined treated the subject of PWPH and long COVID, but did not necessarily look at the same outcomes (see Table 1, under psychiatric evolution evaluation). Looking at Table 2, it seems that the studies evaluating cognitive outcomes (1, 6–8) show more ambiguous results. The only one showing an impact on cognitive function of the COVID-19 infection at follow-up is study number 7, a case report. Studies 1, 6, and 8 show no impact of premorbid psychiatric history at all when measures are adjusted for other risk factors.

Studies concerned specifically with depression and anxiety (*n*°3, 4, and 9) also lack consensus: studies *n*°4 and 9 show similar outcomes for PWPH than for patients without any prior psychiatric comorbidities, and study *n*°3 concludes to a less favorable evolution for PWPH in terms of length of illness.

The studies that looked at neuropsychiatric outcomes with a more global point of view showed more consistency in results, with a trend toward a higher pathological level of results of psychometric testing for PWPH (*n*°2, 10) or a worse persistence of depression (*n*°11) in PWPH than in patients with no prior psychiatric illness.

The study focusing on eating disorders showed that the trigger changed for cravings, having an impact on the control of the disease for the patient (with more cravings, “shifting to habitual behavior”). This was a case report reporting only on one particular patient.

Table 4 summarizes the modality of retrieving patients’ psychiatric history in the different studies and the outcomes related to the studies, categorized by type of assessment (self-reports in questionnaires or interviews versus retrieval from medical records). The majority (7/9) used a self-reporting strategy. The two case reports (*n*°5, 7) included in the review were excluded from these numbers because they did not provide comparisons with patients without psychiatric history.

**Table 4:** Mode of assessment of patient’s prior psychiatric history and outcomes related.

Self-report			Medical records	
Studies 3, 4, 6, 9, 10, and 11; *n* = 7/9 (66.7%)			Studies 1, 2, and 8; *n* = 3/9 (44.4%)	
*Worse outcome for PWPH*	*No effect*	*Inconclusive*	*Worse outcome for PWPH*	*No effect*
*n* = 3 (42.9%) #3; 10; 11	*n* = 2 (28.6%) #4; 9	*n* = 1 (14.3%) #6	*n* = 0 (0%)	*n* = 3 (100%) #1; 2; 8

It seems that studies with a self-report system tend to show outcomes for PWPH that are worse than for patients without prior psychiatric morbidities (42.9%) than studies with a medical record retrieval system (0%).

## 4. Discussion

### 4.1. The impact of prior mental health issues on neuropsychiatric outcomes of PASC

This scoping review has the purpose of evaluating the possible consequences on mental health of long COVID in patients with prior psychiatric history.

Our results show a majority of studies finding a correlation between having a psychiatric history and a worse course of long COVID, with longer recuperation times or worse outcomes than for previously healthy patients. This is especially the case for studies looking at broader neuropsychiatric outcomes.

Nevertheless, a heterogeneity subsists between conclusions drawn on the effects of long term infection for PWPH by the different studies we examined. This is corroborated by the results of Efstathiou et al. (20), who did a thorough search of the literature in a review on neuropsychiatric consequences of COVID-19, occasionally looking at PWPH without being able to find consistent information on a possible association of this psychiatric history to more severe or longer lasting repercussions. We think the main explaining factor is the very few studies regarding this precise topic of research. As can be seen in our PRISMA chart (Figure 1), in the vast majority of studies focusing on mental health and SARS-CoV-2, having a prior psychiatric history was an exclusion criterion, as the subject of research was frequently new-onset neuropsychiatric outcomes in long COVID. This heuristic can be understood as it probably permits avoiding confounding factors that would occur if this population was not separated from patients without any psychiatric history whatsoever. However, it ends up neglecting the psychiatric population, even though it is the most at-risk of developing new symptoms or relapsing into old ones, and should therefore benefit from a higher degree of concern on this matter (21, 22). Even in the studies that we use in our review, PWPH are not always treated as belonging to a separate category and are sometimes mixed with patients having had neurological problems, sometimes with a low rate of PWPH contained in those studies. The population is therefore sometimes very small if we take the articles independently.

None of the studies specified how and if the patients were treated for the mental health disease they had encountered in their life, or if the disease was ongoing. This point makes interpretation of the results delicate. In fact, we can safely assume that undergoing some form of psychiatric treatment would make a patient more prepared to face the mental health issues related to their infectious disease than untreated patients. A patient taking antidepressants at the time of infection because of pre-existing depression or seeing a psychotherapist two times a week during their COVID-19 infection and illness might be protected against negative outcomes of the infection. This could be a piece of field research for further investigation. This is even more interesting since Hoertel et al. (23) suggest in their research that medication by antidepressants could be linked to better recovery from the infectious disease, without any regard to the psychiatric state of patients, by interfering with the disease on several molecular pathways. Gildbody et al. (24) further argue that the studies regarding mental health are almost always observational, and only few clinical trials exist, thus describing the situation without real solutions being researched.

### 4.2. Multiplicity of diverse psychometric scales to measure neuropsychiatric outcomes

The high variety of psychometric scales used was unsettling, especially as the use of one psychometric scale over another was not justified in the respective articles. Clinical interviews to further assess mental health were only conducted in approximately a third of studies, even though it is known to increase the sensitivity and specificity of screening (25). Using only standardized tests has the benefit of being an objective, reproducible way of evaluating someone’s clinical state but it is also probable that it shows an incomplete picture, especially when focusing only on one or two outcomes. Furthermore, when there is such a variety of tests used, it makes the concordance of the results difficult to interpret. Even when two studies agree, the modality of tests is very variable and therefore not as reliable as it would have been if only one scale had been used. Sampogna et al. (26) also find this heterogeneity in the methodology when studying also psychiatric outcomes of COVID infection. It is thus probable that studies focusing on only one psychiatric outcome might miss other outcomes that would be above the clinical threshold if tested.

### 4.3. Treatment of patients

The proportion of patients who were offered treatment were also very low and the treatment consisted exclusively of pharmacotherapy [selective serotonin reuptake inhibitors (SSRI)/selective noradrenaline reuptake inhibitors, off-label modafinil, and treatment by hypnotic drugs]. The effects of treatments were not discussed in the articles selected for the review, but Mazza et al. (27) published an article on the effectiveness of SSRI in post-COVID depression, showing a quicker and better medical response to standard pharmacological treatment compared to non-COVID depression, even in groups of patients with a psychiatric history. The treatment should not be overlooked, as research shows that the rate of depression and anxiety, for instance, has a tendency to slightly increase with time, even in patients without psychiatric history, and therefore not to have a positive spontaneous evolution (28).

### 4.4. Determining psychiatric history

The methods of evaluating who is and who is not a PWPH were either self-report or retrieving of medical records. Self-report has the advantage of being highly sensible, for instance allowing patients with anxiety and depression that do not seek medical support to be also included in the cohort of PWPH. However, it is also less precise. The retrieval of medical records is probably more specific, but it could miss a lot of mentally at-risk patients who did not consult a doctor for their issues. Table 4 reveals a difference between the results depending on the way of determining psychiatric history—none of the studies with a medical record retrieval system show an effect of prior psychiatric history on mental health outcomes in long COVID, when almost half of the self-report system seems to show conclusive correlation. This can be explained in a variety of ways, one being a small sample size of included studies. But one could also argue that when retrieving medical records, the threshold to detect mental illness is significantly higher than when asking patients how they feel, missing a population of at-risk patients but not clinically diagnosed. It is known, for instance, that patients with a prior depressive episode, even a mild to moderate one, for which patients do not always see doctors, tend to relapse and these chances of occurrence increase with the number of episodes (29). Retrieval from medical records could therefore miss some part of the target population and may bias the results toward patients benefiting from a better follow-up and treatment (23).

### 4.5. Weaknesses of the scoping review

In order to preserve study quality, this review excludes some categories of neuropsychiatric outcomes that are more often and precisely studied than the ones reviewed, such as sleep disorders, alcohol abuse, erectile dysfunction, and gambling problems.

The geographic distribution of the articles selected is sub-optimal, as almost the totality of the studies show data regarding Europe (Austria, Belgium, and Italy) and Northern America (Canada, United States) because we could not find studies based elsewhere (Africa, South America, and Oceania) that corresponded to the inclusion criteria. Only one Asian study (Japan) was included. Even in Europe and Northern America, the geographic distribution is not representative, as four of the studies are based in Milan, Italy. This is due to a current lack of publication in other countries, for reasons that are unknown but might be an institutional interest bias, a lack of funding, or other causes.

### 4.6. Strength of the scoping review

The strength of this scoping review lies mainly in highlighting the lack of evidence and research on the outcomes in mental health in the vulnerable population of patients with prior mental health problems. Since it is now recognized that long COVID can impact mental health, even on previously healthy patients, it is surprising that so few studies exist even though we can suspect their results to be interesting.

To our knowledge, it is also the first review that assesses the consequences of long COVID in this at-risk population of patients and some of the data seem to preliminarily show that there is an impact of previous mental disorder or experiencing mental health adversity in recovery from long COVID, either in an increase of severity of psychiatric symptoms or in a longer convalescence time.

This scoping review should therefore increase the interest of the authorities and make doctors more alert to the risk of mental disorders after COVID, not only in healthy people but also in patients known for psychiatric history as well.

### 4.7. Conclusion

In this scoping review, we looked at studies regarding long COVID and its effects on the mental health of patients burdened with psychiatric illnesses predating their SARS-CoV-2 infection.

Some of the data seem to indicate that patients with a history of psychiatric illness may experience worse effects of long COVID than previously healthy patients and may need a longer recovery time. However, these data are preliminary and need to be confirmed by other studies.

Long COVID is a field of active research, with some areas, such as the psychiatric aspects, not being completely elucidated. The present research highlights how the psychiatric population is neglected in the yet so-rich state of current research regarding SARS-CoV-2 and long COVID. It shows the lack of studies specifically dedicated to the qualitative and quantitative aspects of mental health in the most at-risk population. Until definitive results determine if there is or is not an effect of prior psychiatric history on response to psychiatric impact of the disease, we should definitely treat this population carefully and be alert to any distress manifestations.

## Author contributions

AF, FH, and AD: conceptualization and methodology. AF, LM, AD, and AB: validation. FH and AB: formal analysis. FH: investigation and writing—original draft preparation. FH, AD, AB, LM, and AF: resources and writing—review and editing. All authors contributed to the article and approved the submitted version.

## Funding

Open access funding was provided by University of Geneva.

## Conflict of interest

The authors declare that the research was conducted in the absence of any commercial or financial relationships that could be construed as a potential conflict of interest.

## Publisher’s note

All claims expressed in this article are solely those of the authors and do not necessarily represent those of their affiliated organizations, or those of the publisher, the editors and the reviewers. Any product that may be evaluated in this article, or claim that may be made by its manufacturer, is not guaranteed or endorsed by the publisher.

## Appendix

Appendix A: Research queries

- ***for PubMed:*** (“post-acute COVID-19 syndrome”[Supplementary Concept] OR “post-acute sequelae of COVID-19”[Title/Abstract] OR “Post-COVID-19”[Title/Abstract] OR “Covid 19/complications”[MeSH Terms] OR “post Covid syndrome”[Title/Abstract] OR “long term Covid”[Title/Abstract] OR “long Covid”[Title/Abstract] OR “post-acute Covid”[Title/Abstract] OR “post Covid”[Title/Abstract] OR “long haul Covid”[Title/Abstract] OR “long hauler Covid”[Title/Abstract] OR “persistent Covid”[Title/Abstract] OR “Covid-19-related”[Title/Abstract]) AND (“Mentally Ill Persons”[MeSH Terms] OR “Mental Disorders”[MeSH Terms] OR “psychiatric history”[Title/Abstract] OR “mental illness”[Title/Abstract] OR “psychiatric comorbidities”[Title/Abstract] OR “mental health patient*”[Title/Abstract] OR “psychiatric history”[Title/Abstract] OR “psychiatry consultation”[Title/Abstract] OR “outpatient psychiatric”[Title/Abstract] OR “mentally ill person*”[Title/Abstract] OR “mental patient*”[Title/Abstract] OR “psychiatry patient*”[Title/Abstract] OR “psychiatric patient*”[Title/Abstract] OR “pre-existing depression”[Title/Abstract] OR “neuropsychiatric manifestations”[Title/Abstract] OR “poor mental health”[Title/Abstract])
- ***for Embase:*** (‘long Covid’/de OR ‘post Covid syndrome’:ab,ti,kw OR ‘long term Covid’:ab,ti,kw OR ‘long Covid’:ab,ti,kw OR ‘long term illness in Covid-19’:ab,ti,kw OR ‘post-acute Covid’:ab,ti,kw OR ‘post-Covid’:ab,ti,kw OR ‘long-term sequelae of Covid’:ab,ti,kw OR ‘post-acute Covid-19 sequelae’:ab,ti,kw OR ‘Covid-19 long-hauler’:ab,ti,kw OR ‘long haul Covid’:ab,ti,kw OR ‘long hauler Covid’:ab,ti,kw OR ‘persistent Covid’:ab,ti,kw OR ‘post-acute sequelae of Covid-19’:ab,ti,kw OR ‘post-Covid-19’:ti,kw,ab OR ‘sars-cov-2 infection’:ab,ti,kw) AND (‘mental patient’/de OR ‘previous psychiatric history’:ab,ti,kw OR ‘prior psychiatric comorbidities’:ab,ti,kw OR ‘psychiatric comorbidities patient*’:ab,ti,kw OR ‘mental health patient*’:ab,ti,kw OR ‘psychiatric precedent*’:ab,ti,kw OR ‘psychiatric history’:ab,ti,kw OR ‘patients on a psychiatry consultation’:ab,ti,kw OR ‘infection psychosis’:ab,ti,kw OR ‘mentally ill person*’:ab,ti,kw OR ‘mental patient*’:ab,ti,kw OR ‘psychiatr* patient*’:ab,ti,kw OR ‘poor mental health’:ab,ti,kw OR ‘pre-existing depression’:ab,ti,kw OR ‘psychiatric comorbidities’:ab,ti,kw OR ‘neuropsychiatric manifestations’:ab,ti,kw OR ‘psychiatry consultation’:ab,ti,kw OR ‘outpatient psychiatric’:ab,ti,kw OR ‘psychological distress’:ab,ti,kw OR ‘pre-existing depression’ OR ‘history of depressive’:ab,ti,kw) NOT ‘conference abstract’/it

Appendix B: Bibliography of articles used in the scoping review

1. Aiello EN, Radici A, Mora G, Pain D. Cognitive phenotyping of post-infectious SARS-CoV-2 patients. Neurol Sci. août 2022;43(8):4599–604.
2. Fiabane E, Pain D, Aiello EN, Radici A, Manera MR, Grossi F, et al. Psychiatric symptoms subsequent to COVID-19 and their association with clinical features: A retrospective investigation. Psychiatry Res. oct 2022;316:114757.
3. Hazumi M, Usuda K, Okazaki E, Kataoka M, Nishi D. Differences in the Course of Depression and Anxiety after COVID-19 Infection between Recovered Patients with and without a Psychiatric History: A Cross-Sectional Study. Int J Environ Res Public Health. 8 sept 2022;19(18):11316.
4. Hüfner K, Tymoszuk P, Ausserhofer D, Sahanic S, Pizzini A, Rass V, et al. Who Is at Risk of Poor Mental Health Following Coronavirus Disease-19 Outpatient Management? Front Med (Lausanne). 2022;9:792881.
5. Leenaerts N, Ceccarini J, Sunaert S, Vrieze E. The impact of COVID-19-related smell and taste disorders on a patient with bulimia nervosa: a case report. Neurocase. févr 2022;28(1):72–6.
6. Liyanage-Don NA, Winawer MR, Hamberger MJ, Agarwal S, Trainor AR, Quispe KA, et al. Association of depression and COVID-induced PTSD with cognitive symptoms after COVID-19 illness. Gen Hosp Psychiatry. juin 2022;76:45–8.
7. Tolsdorf EL, Gojmerac C, Crowson J, Frey BN, Kapczinski F, Duarte D. Conversion to major neurocognitive disorder after COVID-19 in a woman with bipolar disorder: A 6-year longitudinal case report. Bipolar Disord. juin 2022;24(4):451–6.
8. Whiteside DM, Basso MR, Naini SM, Porter J, Holker E, Waldron EJ, et al. Outcomes in post-acute sequelae of COVID-19 (PASC) at 6 months post-infection Part 1: Cognitive functioning. Clin Neuropsychol. mai 2022;36(4):806–28.
9. Farooqi M, Khan A, Jacobs A, D’Souza V, Consiglio F, Karmen CL, et al. Examining the Long-Term Sequelae of SARS-CoV2 Infection in Patients Seen in an Outpatient Psychiatric Department. Neuropsychiatr Dis Treat. 2022;18:1259–68.
10. Mazza MG, De Lorenzo R, Conte C, Poletti S, Vai B, Bollettini I, et al. Anxiety and depression in COVID-19 survivors: Role of inflammatory and clinical predictors. Brain, Behavior, and Immunity. 1 oct 2020;89:594–600.
11. Mazza MG, Palladini M, De Lorenzo R, Magnaghi C, Poletti S, Furlan R, et al. Persistent psychopathology and neurocognitive impairment in COVID-19 survivors: Effect of inflammatory biomarkers at three-month follow-up. Brain, Behavior, and Immunity. May 2021;94:138–47.
